# How to Write a Presubmission Inquiry

**DOI:** 10.1371/journal.pcbi.1004098

**Published:** 2015-02-26

**Authors:** Thomas Lengauer, Ruth Nussinov

**Affiliations:** 1 Department of Computational Biology and Applied Algorithmics, Max Planck Institute for Informatics, Saarbrücken, Germany; 2 Cancer and Inflammation Program, Leidos Biomedical Research, Inc., Frederick National Laboratory for Cancer Research, National Cancer Institute, Frederick, Maryland, United States of America; 3 Sackler Institute of Molecular Medicine, Department of Human Genetics and Molecular Medicine, Sackler School of Medicine, Tel Aviv University, Tel Aviv, Israel

Like many other journals, the journal *PLOS Computational Biology* admits and in some cases requires presubmission inquiries to be submitted before the submission of a full paper. Presubmission inquiries serve the purpose of informing the journal’s Editorial Board of the essence of the intended submission. Based on the information in the inquiry, the editors can make a quick assessment of its contribution with respect to the criteria for publication in the journal. This assessment is then communicated to the authors. This enables fast turnaround to the authors about the basic suitability of a submission for processing by the journal and spares the editors and reviewers the effort of detailed inspection of submissions that clearly do not meet the criteria of the journal.

In this Editorial, we give suggestions for preparing presubmission inquiries for journal submissions. We exemplify these suggestions with reference to presubmission inquiries for the Methods section of *PLOS Computational Biology*. However, our suggestions generalize to presubmission inquiries of other kinds and for other journals, and in places, we will make specific comments to that effect.

Over two years ago, *PLOS Computational Biology* opened a special section dedicated to Methods papers. As the scope statement spells out,

*Methods papers should describe outstanding methods of exceptional importance that have been shown, or have the promise to provide new biological insights. The method must already be widely adopted, or have the promise of wide adoption by a broad community of users. Enhancements to existing published methods will only be considered if those enhancements bring exceptional new capabilities*.

Since Methods papers are different from other research papers in *PLOS Computational Biology* and also differ from typical papers on bioinformatics methods published in other journals, a mandatory presubmission stage has been introduced for the submission of Methods papers to *PLOS Computational Biology*. (Note that a presubmission inquiry is not mandatory for general research papers in *PLOS Computational Biology*, though it is also mandatory for submission to the Software papers category.)

Since the Methods section was launched in October 2012, we have received 334 presubmission inquiries. For roughly half of them (159), we encouraged submission and received full papers, of which 41 papers were published, so far, as Methods papers. We find that, while many presubmission inquiries are informative enough to make an educated decision on the submission, we also receive a number of presubmission inquiries that are not sufficiently informative, such that submission may be discouraged not on the basis of the quality or scope of the paper but on that of the presubmission inquiry. We generally do not allow revisions of presubmission inquiries. In order to minimize the number of papers that fail to get a chance to be published in *PLOS Computational Biology* merely because of the inadequacy of the presubmission inquiry, here we give a number of suggestions for preparing such an inquiry.

The goal of a presubmission inquiry is to make the statement to the Editorial Board that the paper to be submitted reasonably satisfies the criteria detailed in the scope statement. The presubmission inquiry must be detailed enough to convincingly make that point. Most of the insufficiently informative inquiries that we get are either too terse, i.e., they do not give enough detail, or they are not specific enough. Therefore, we suggest a way of structuring a presubmission inquiry. These suggestions are the result of our experience with presubmission inquiries over the past couple of years.

1. **What is the problem?** Please summarize the problem domain and statement and the relevance of the problem to the general readership of the journal—in the case of *PLOS Computational Biology*, the biological research community or a substantial subcommunity. What is that subcommunity? How relevant is the problem to them?
2. **What is the innovation?** Here it is important that you give enough detail on your contribution to allow the Editorial Board to form an image of the substance and relevance of the advance over the state of the art in the field and over your previous work. If your paper presents material that rests on or is related to your own previous publications, this entails addressing dual publication issues. In order to argue your point, you have to summarize the state of the art on which you base your contribution and give the essential ingredients of your innovation. Depending on the journal, the innovation can take different shapes: a contribution to technology or experimental design, a biological finding, a methodical or theoretical piece of work, etc. For papers in the Methods section of *PLOS Computational Biology*, the computational method is expected to be at the center of the innovation. This section is not for papers whose methodical core has been published elsewhere and for which you present a—possibly extended or modified—application scenario. Also, studies presenting a comparative assessment of existing methods on an application domain are not within the scope of a Methods paper. We expect a concrete and specific relationship to underlying biological issues. This is why general methods on statistical learning that find their application in biology as well as in other fields of science are typically not considered in scope, unless the paper focuses on sufficiently deep issues of the configuration of the method that are specific to biology. Finally, the method must be the major innovation of the paper. However interesting it may be, a biological finding that has been obtained with methods that are prepublished or only minor modification of prepublished methods is not within the scope of the Methods papers category. (On the other hand, it may be a suitable General research paper for the journal.)
3. **How is the method validated?** Validation can take manifold forms but is a key element in most scientific papers. For a theoretical paper, the validation often takes the form of a proof. In contrast, methods in computational biology have manifold forms of validation and, usually, a single form is not sufficient to make the point. For instance, for the Methods section of *PLOS Computational Biology*, we expect more than an anecdotal validation based on a couple of biological use cases. A validation purely on synthetic data is not sufficient either. Rather, the validation must make a convincing argument for the general applicability of the method in a substantial biological problem domain. Please note, however, that papers that center on the validation of a method that has been published elsewhere are not considered in scope either. The paper has to contain both the method and its application.
4. **How is the method being made available?** Availability of research results becomes an increasingly desired and often required aspect of a publication. For papers that are based on experimental data, making the data and the protocols of the experimental design available is a prerequisite for making the research reproducible. For methods papers, reusability of the method also becomes an issue. For the Methods section of *PLOS Computational Biology*, we only accept papers on methods that are useful to and can be readily applied by other scientists. The best way of satisfying this criterion is to make the software implementation of the methods openly available. For methods that are not based on software, a workable protocol for how to use the methods must be provided.

If you have the full submission ready at the time of the presubmission inquiry, we encourage you to attach it to the inquiry as an optional supplement and mention that you have done this in your cover letter. However, your presubmission inquiry should be worded such that the editors do not need to inspect the complete paper for making their assessment.

We wish you much success with your future submission to the Methods section of *PLOS Computational Biology*.

